# CT‐Imaging Manifestations and Diagnostic Insights in Pulmonary Intravascular Large B‐Cell Lymphoma: A Case Series and Literature Review

**DOI:** 10.1002/cnr2.70159

**Published:** 2025-02-28

**Authors:** Xinyi Gou, Yinli Zhang, Yuan Li, Libao Hu, Jin Cheng, Nan Hong

**Affiliations:** ^1^ Department of Radiology Peking University People's Hospital Beijing China; ^2^ Department of Pathology Peking University People's Hospital Beijing China; ^3^ Department of Nuclear Medicine Peking University People's Hospital Beijing China; ^4^ Department of Thoracic Surgery Peking University People's Hospital Beijing China

**Keywords:** chest computed tomography, ground‐glass opacities, intravascular large B‐cell lymphoma

## Abstract

**Background:**

Intravascular large B‐cell lymphoma (IVLBCL) is a rare and aggressive subtype of non‐Hodgkin lymphoma. Although autopsy findings have revealed that IVLBCL often involves the lungs, its clinical features and imaging manifestations have rarely been reported. This study aimed to explore the pulmonary imaging manifestations of three patients with pulmonary IVLBCL.

**Case:**

We retrospectively reviewed the clinical data and chest computed tomography (CT) images of three patients diagnosed with pulmonary IVLBCL between January 2010 and July 2023 in our hospital. In this case series, three patients (2 males and 1 female, aged 51–65 years) presented with a variety of symptoms. Laboratory tests revealed elevated lactate dehydrogenase levels in all three individuals. Chest CT scans revealed bilateral ground‐glass opacities (GGOs) in the lungs in all cases. Although previous case reports have often depicted GGOs as ill‐defined, two of the three cases manifested multiple well‐circumscribed GGOs. Two cases underwent fluorodeoxyglucose positron emission tomography (FDG) PET/CT scans, which showed increased FDG uptake in pulmonary lesions (SUVmax values of 1.9 and 1.7, respectively).

**Conclusion:**

Chest CT images of patients with pulmonary IVLBCL mainly manifested as bilateral GGOs of the lungs, with ill‐defined GGO infiltrations being more common. FDG PET/CT demonstrates high diagnostic sensitivity for detecting pulmonary involvement in IVLBCL. Even when chest CT scans reveal no visible abnormalities, FDG PET/CT can detect enhanced metabolic uptake in areas with lesions for certain patients.

**Abbreviations**
BALF: bronchoalveolar lavage fluid
FDG PET/CT: fluorodeoxyglucose positron emission tomography/computed tomography
GGNs: ground grass nodules
GGOs: ground‐glass opacities
IVLBCL: Intravascular large B‐cell lymphoma
LDH: lactate dehydrogenase
TBLB: transbronchial lung biopsy

## 1 Background

Intravascular large B‐cell lymphoma (IVLBCL) is a rare subtype of non‐Hodgkin lymphoma, characterized by the filling of lymphoma cells in the vascular lumen [1]. IVLBCL occurs infrequently, with an estimated prevalence of about 0.095 per million people in the United States and roughly 0.5 cases per million worldwide [2, 3].

It has a heterogeneous presentation with no lymphadenopathy or detectable lymphoma cells in the peripheral blood. IVLBCL has a slight male predominance of 1.1:1 and predominantly affects the elderly, with a median age at diagnosis of 67 years (range, 41–85 years) [4]. It is highly invasive and can involve various organs throughout the body, and pulmonary involvement is frequently observed in autopsy studies [5, 6]. Its prognosis is extremely poor, with a median survival time of approximately 1 year [7].

Early diagnosis is crucial for IVLBCL patients, as previous studies have shown that patients who receive timely and appropriate treatment have a better prognosis [8]. Due to its rare occurrence and ambiguous clinical presentation (such as fever of unknown origin and dyspnea), IVLBCL is frequently misdiagnosed or only identified during post mortem examination [9, 10, 11]. Laboratory findings in patients with IVLBCL with lung involvement typically include anemia, a low platelet count, and high serum lactate dehydrogenase (LDH) levels [12, 13]. Confirming the diagnosis requires histological examination, typically through a random skin biopsy or bone marrow biopsy and transbronchial lung biopsy (TBLB) [10, 14, 15, 16, 17]. While the path from non‐specific symptoms to histopathological confirmation can be challenging, medical imaging techniques serve a vital role in providing supplementary diagnostic information.

Most patients with pulmonary IVLBCL undergo chest computed tomography (CT) scans because of fever and respiratory symptoms. This may provide an opportunity to suspect IVLBCL. Fluorodeoxyglucose positron emission tomography/computed tomography (FDG PET/CT), which has demonstrated a high sensitivity in detecting non‐Hodgkin's lymphoma, was prominent [18, 19]. Therefore, further understanding of the imaging findings of IVLBCL with pulmonary involvement is crucial for early diagnosis and prognosis improvement [12].

Although previous studies have reported that ground‐glass opacities (GGO) and diffuse FDG uptake can be seen on imaging of lung involvement, the radiological features are still not well described. The present study aimed to provide a detailed descriptive evaluation of radiological findings associated with pulmonary IVLBCL. Through an analysis of three case studies and a review of published reports, we sought to characterize the imaging patterns that can aid in recognizing this rare disease, thereby enhancing the diagnostic accuracy and guiding clinical management.

## 2 Case Presentation

All procedures performed in studies involving human participants were in accordance with the ethical standards of the Helsinki Declaration (as revised in 2013), and the study was approved by the Ethics Review Committee of our hospital. We retrospectively reviewed the clinical data and chest images of three patients diagnosed with pulmonary IVLBCL at Peking University People's Hospital between January 2010 and July 2023. The inclusion criteria were as follows: (1) diagnosis of IVLBCL based on pulmonary pathology samples, and (2) availability of corresponding chest CT images. The clinical information and chest CT imaging manifestations of these patients are summarized in Table 1.

**TABLE 1:** Clinical characteristics and examination findings of the three patients.

	Case 1	Case 2	Case 3
Age	65	63	51
Sex	Male	Female	Male
Involving site			
Pulmonary	+	+	+
Brain	−	−	+
Other	−	−	+
Clinical features			
Fever	−	−	+
General fatigue	+	−	+
Laboratory examination			
LDH (U/L)	\	338	1250
RBC (10^12^/L)	4.22	3.99	4.6
WBC (10^9^/L)	4.5	5.7	6.7
Plt (10^9^/L)	155	254	76
Hemoglobin (g/L)	128	123	157
Ki‐67 (%)	90	90	10
Ground‐glass opacities	+	+	+
With consolidation	+	−	−
Ill‐Defined GGOs	−	−	+
Associated findings			
Pleural effusion	−	−	+
Pericardial effusion	−	−	−
Interlobular septal thickening	−	−	−
Distribution			
Axial plane	Random	Peripheral	Random
Coronal plane	Middle and upper lung field predominant	Upper lung field predominant	Diffuse

### 2.1 Case 1

**FIGURE 1:**
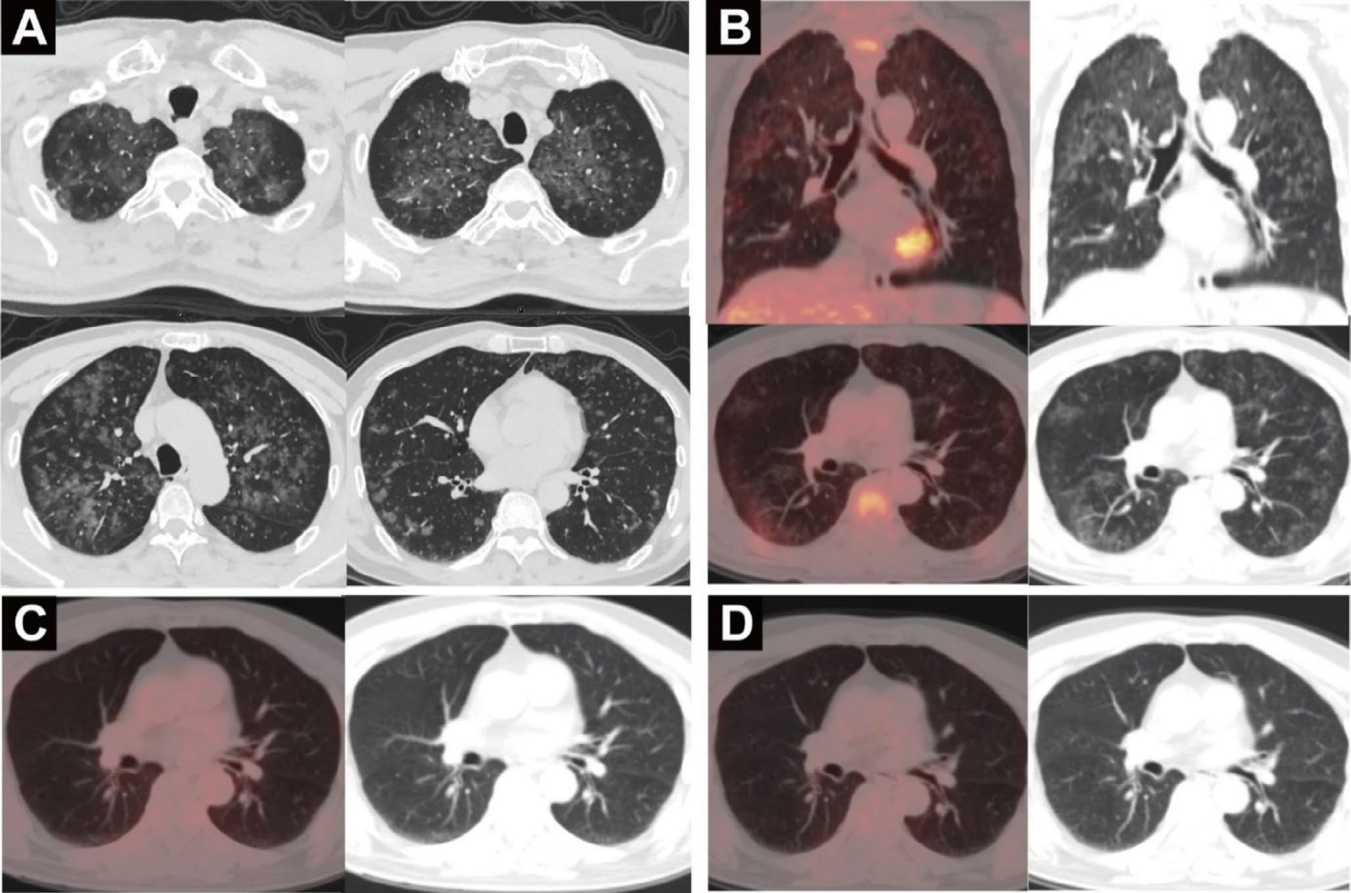
(A) Chest CT images before chemotherapy showing bilateral diffuse patchy and nodular GGOs of the lungs; (B) FDG PET/CT images before chemotherapy showing increased FDG uptake; FDG PET/CT after four (C) and eight (D) rounds of chemotherapy showing that previous lesions disappeared and no FDG uptake was observed.

**FIGURE 2:**
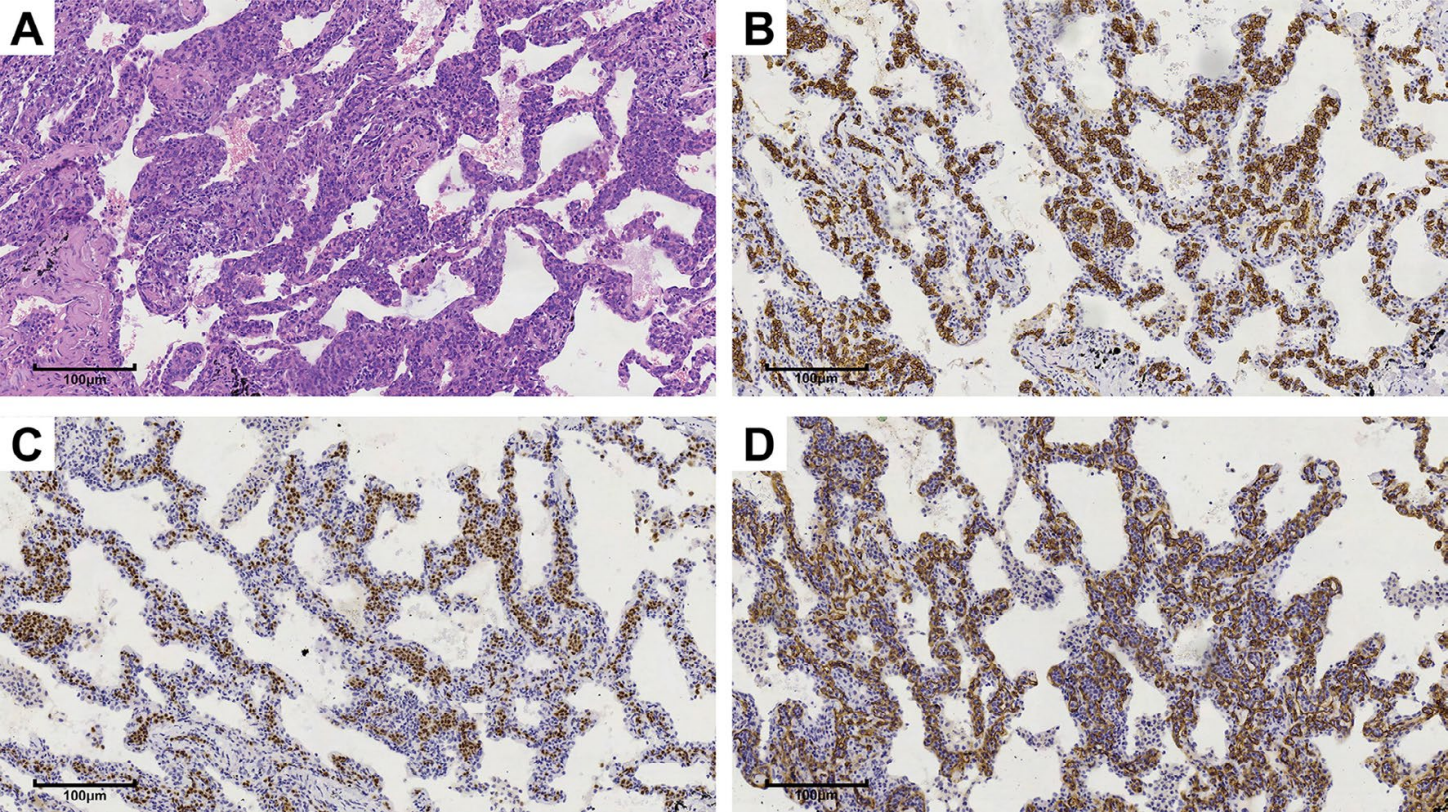
Lung wedge resection specimen (right middle lobe), (A) hematoxylin and eosin staining, ×20 magnification; (B) immunohistochemical staining for CD20, ×20 magnification; (C) PAX5 staining, ×20 magnification; (D) immunohistochemical staining for CD34, ×20 magnification.

A 65‐year‐old male was admitted to our hospital in April 2019 because of a month history of paroxysmal dry cough, dyspnea, and fatigue. Diffuse patchy and nodular GGOs were found in both lungs, mostly in the middle and upper lung fields, with clear subpleural areas (Figure 1A). An FDG PET/CT scan (April 23, 2019) showed that these lesions were accompanied by increased FDG uptake with an SUVmax of 1.9 (Figure 1B). After thoracoscopic lung wedge resection, multiple nodular lesions were observed in the lung tissue of the right middle lobe specimen; the alveolar septa were widened in the lesion area, the capillaries were dilated, and medium to large lymphoid cells were observed in the lumen, with fine chromatin and small nucleoli (Figure 2). Immunohistochemical staining showed CD20(+), CD34(−), CD3(−), CD43(−), PAX‐5(+), CD30(−), bcl‐6(−), mum‐1(+), CD10(−), and a Ki‐67 proliferation index of > 90%. Therefore, the diagnosis of pulmonary IVLBCL was confirmed.

The patient underwent four cycles of R‐CHOP chemotherapy (rituximab, cyclophosphamide, epirubicin, vinesine, and dexamethasone). FDG PET/CT re‐examination (August 19, 2019) showed that the bilateral diffuse GGOs had disappeared and no obvious FDG uptake was observed, indicating a complete metabolic response (Figure 1C). After four additional cycles of R‐CHOP chemotherapy and rituximab monotherapy consolidation treatment, a follow‐up FDG PET/CT revealed continued complete metabolic response on January 2, 2020 (Figure 1D). No recurrence was observed during the approximately three‐year follow‐up period ending on January 20, 2022.

### 2.2 Case 2

**FIGURE 3:**
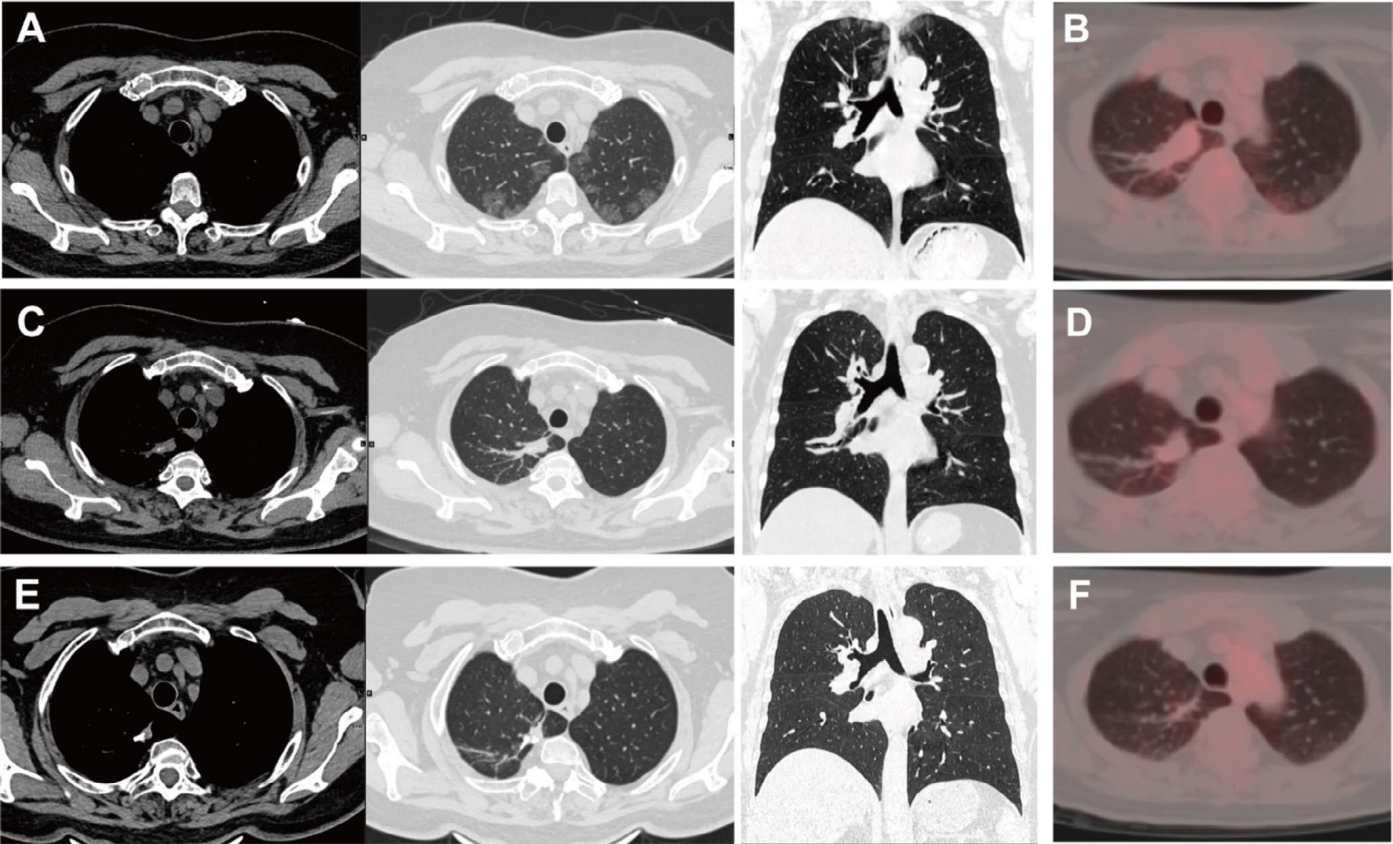
(A) Chest CT images before chemotherapy showing multiple GGOs beneath the pleura in the bilateral upper lobes of the lungs; (B) FDG PET/CT images before chemotherapy; (C, D) chest CT images and FDG PET/CT images after the first round of chemotherapy showing disappearance of lesions and no FDG uptake; (E, F) chest CT images and FDG PET/CT images after the second round of chemotherapy showing disappearance of the lesions and no FDG uptake.

**FIGURE 4:**
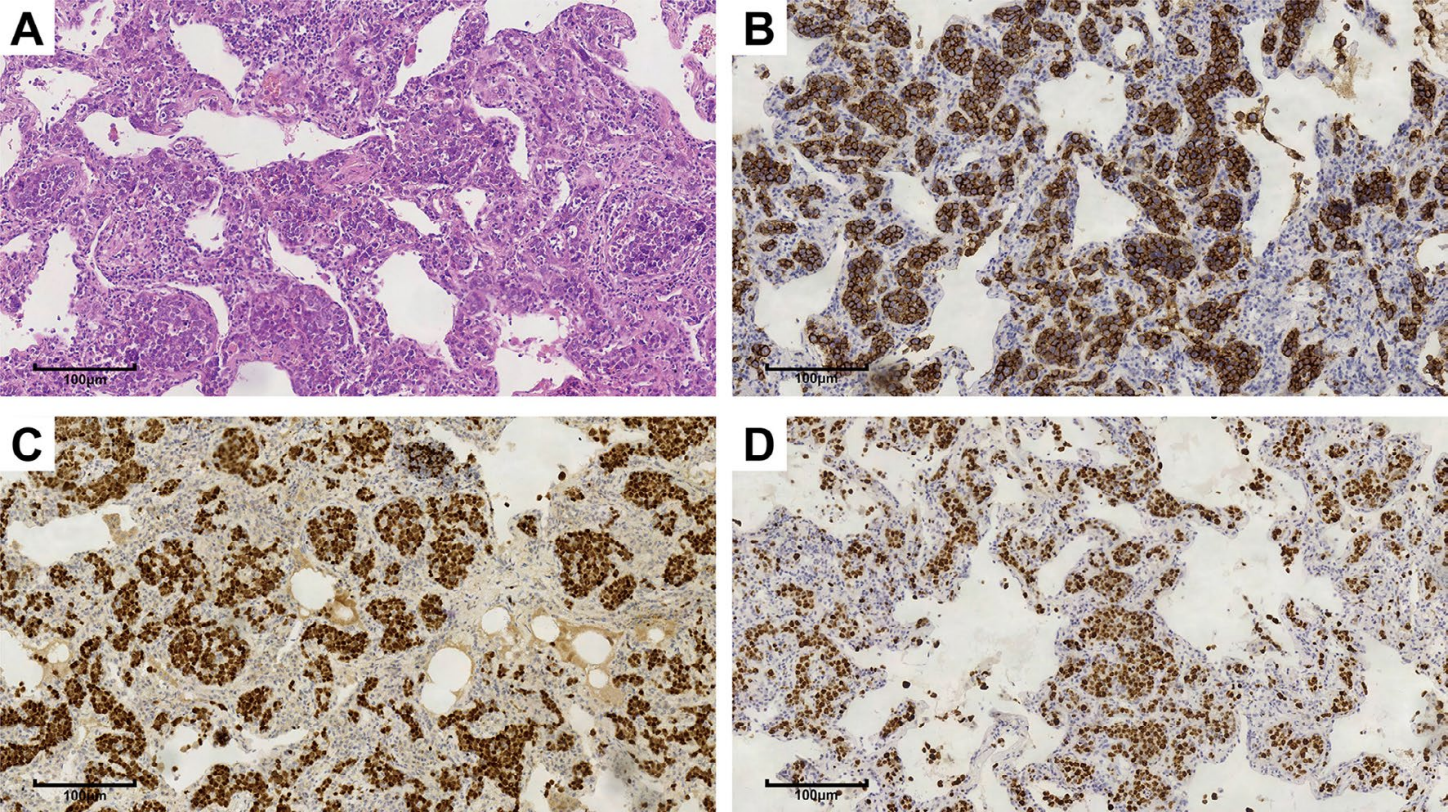
Lung wedge resection specimen (right upper lobe), (A) hematoxylin and eosin staining, ×20 magnification; (B) Immunohistochemical staining for CD20, ×20 magnification; (C) PAX5 staining, ×20 magnification; (D) Ki‐67 staining, ×20 magnification.

A 64‐year‐old woman with multiple ground‐glass nodules (GGNs) detected during a medical checkup was referred to our hospital on August 12, 2020. She had no respiratory symptoms, and after a 12‐day course of antibiotics, no significant changes were observed in her lung condition. During outpatient follow‐up on October 28, 2020, chest CT images revealed multiple well‐defined round GGNs (the largest one was approximately 6 mm) beneath the pleura in the bilateral upper lobes of the lung (Figure 3A). TBLB and bronchoalveolar lavage fluid (BALF) analysis were performed (November 2, 2020), but no significant abnormalities were found. The patient underwent FDG PET/CT (November 10, 2020) after TBLB, which revealed multiple GGNs and increased FDG uptake in some areas (SUVmax: 1.7) (Figure 3B). After wedge resection by thoracoscopy on November 11, 2020, pathological examination showed that the lung tissue had interstitial vascular dilation and was filled with lymphoid cells of moderate size with nuclear atypia, visible nucleoli, and mitotic figures (Figure 4). Immunohistochemically, these atypical cells were positive for CD20, CD5, PAX‐5, BCL2, BCL6, and MUM1; negative for CD3, CD5, and CD10; and had a Ki‐67 proliferation index of > 90%. The patient was diagnosed with pulmonary IVLBCL.

After one round of chemotherapy with the R‐CHOP regimen, chest CT images (January 15, 2021) revealed that the GGNs had disappeared (Figure 3C,E), and there was no metabolic activity on FDG PET/CT (February 9, 2021) (Figure 3D,F). Subsequently, complete metabolic response was considered. The patient received R‐CHOP regimen chemotherapy and rituximab monotherapy in the following 1 month. Continuous follow‐up monitoring was then conducted. The latest chest CT scan on February 5, 2025, did not reveal any significant abnormalities, and no recurrence has been observed.

### 2.3 Case 3

**FIGURE 5:**
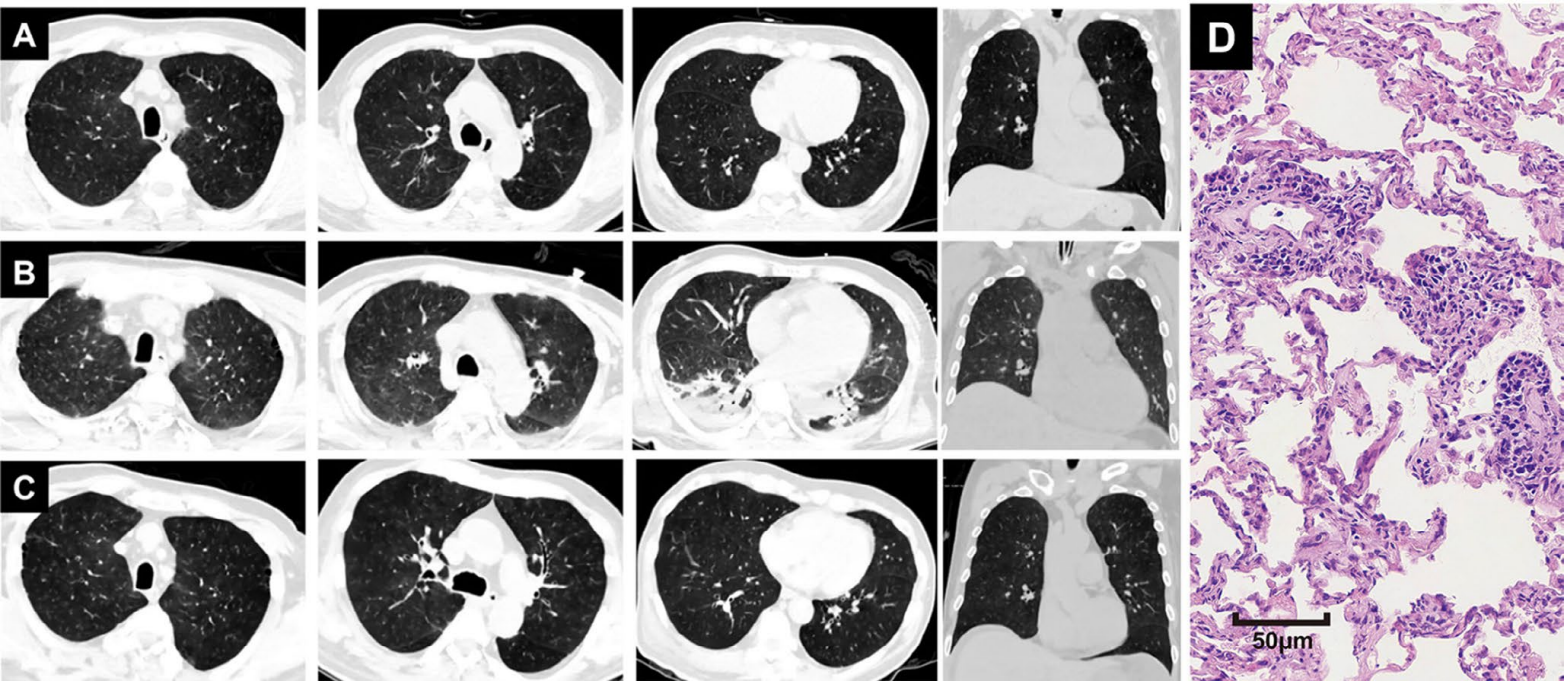
(A) Baseline chest CT images revealed bilateral diffuse ground‐glass infiltrates; (B) following empirical treatment, chest CT images demonstrated persistent diffuse and hazy ground‐glass opacities in both lungs, accompanied by pleural effusion; (C) after immunoglobulin and hormone therapy, chest CT images showed diffuse GGOs in both lungs, which had improved, and a small amount of pleural effusion on both sides had been absorbed. (D) Hematoxylin and Eosin staining of the lung biopsy specimens (×20 magnification).

A 51‐year‐old man presented with dyspnea for 2 months on May 19, 2022. Laboratory results showed elevated LDH, decreased platelet count, and decreased albumin levels. And C‐reactive protein was 18.5 mg/L. Chest CT images (May 20, 2022) showed bilateral diffuse ground‐glass infiltration with unclear boundary (Figure 5A). Empiric treatment with anti‐infection and symptomatic treatments was ineffective. Chest CT images (May 30, 2022) showed diffuse and hazy ground‐glass infiltrates in both lungs and pleural effusion (Figure 5B). Hormone and immunoglobulin therapies were administered, and the symptoms improved. Moreover, chest CT (June 17, 2022) showed diffuse GGOs in both lungs, which had improved compared with before, and a small amount of pleural effusion on both sides was absorbed compared with before (Figure 5C). The lung biopsy sample from the outer basal segment of the right lower lobe showed that the alveolar septum was widened and the inner vascular wall was filled with moderately large lymphoid cells, revealing intravascular large B‐cell lymphoma in the lungs. Pulmonary IVLBCL was confirmed. Unfortunately, the patient was discharged from the hospital against medical advice, and death was confirmed 3 months later via telephone follow‐up.

## 3 Discussion

IVLBCL is characterized by the preferential growth of malignant lymphocytes within the lumina of small vessels. In cases with isolated pulmonary involvement, tumor cells infiltrate the pulmonary vasculature, resulting in local ischemia and inflammation. This can manifest as symptoms such as dyspnea and cough, accompanied by specific radiological abnormalities. It typically has a poor prognosis and is often fatal. Although early and standardized chemotherapy can greatly improve the prognosis of patients with IVLBCL, early diagnosis remains a major challenge due to the nonspecific clinical presentation and low incidence of this disease. This study reported three cases of pulmonary IVLBCL (Table 1) and reviewed chest CT images and clinical information of 32 patients with pulmonary IVLBCL in previous case reports (Tables 2 and 3).

**TABLE 2:** Summary of reported IVLBCL with pulmonary involvement and with abnormal chest CT findings.

References	Case	Age	Sex	Clinical findings			Laboratory findings			CT findings
				Fever	Cough	Dyspnea	LDH	CRP	sIL2‐R	
[20]	No. 1	35	F	37°C–39°C	Yes	Yes	1554 IU/L	175 U/L	NA	Bilateral interstitial shadows with small granules and nodules
[21]	No. 1	54	F	37.6°C	Yes	Yes	749 IU/L	NA	NA	Bilateral diffuse, hazy ground‐glass infiltrates throughout the lungs
[22]	No. 1	69	F	37.3°C–39.8°C	Yes	Yes	2250 IU/L	88 mg/L	NA	Increased attenuation in bilateral lung parenchyma with multiple GGOs and part progression to consolidation, especially on the superior lobes
[22]	No. 2	68	M	Low‐grade fever	Yes	Yes	1449 IU/L	12 mg/L	NA	A ground pattern in a mosaic distribution and small centrilobular nodules
[22]	No. 4	60	F	Intermittent high fevers to 39°C	NA	Yes	1542 IU/L	80 mg/L	NA	Pulmonary nodules with part‐solid diffused GGOs in the lungs without pleural involvement
[23]	No. 1	70	F	Intermittent high fevers to 39°C	Yes	Yes	1977 U/L	NA	NA	Patchy GGO throughout both lungs with lower‐lobe predominance
[24]	No. 1	56	M	Yes	NA	Yes	1416 U/L	NA	1744 U/mL	Bilateral diffuse faint GGOs
[25]	No. 1	73	M	Yes	Yes	Yes	562 U/L	17.9 mg/dL	8313 U/mL	Diffusely spreading GGOs in both lungs
[26]	No. 1	76	F	NA	NA	Yes	NA	NA	NA	Diffuse interstitial thickening and GGOs with a basilar predominance within the lungs
[27]	No. 1	44	M	Yes	Yes	Yes	1000 U/L	NA	NA	Diffuse hazy ground glass infiltrates throughout both lung fields
[28]	No. 1	75	M	NA	NA	NA	1491	39.8	NA	Bilateral GGOs predominantly in the upper lobes with increased nodularity
[29]	No. 1	59	M	No	Yes	No	712 U/L	NA	NA	Bilateral patchy GGOs and reversed halo sign
[30]	No. 1	74	M	NA	NA	Yes	238 U/L	0.6 mg/dL	1277 U/mL	Multiple bilateral pulmonary GGNs
[31]	No. 1	39	F	No	Yes	Yes	1532 U/L	35.39 mg/L	NA	Increased attenuation in bilateral lung parenchyma with multiple GGOs
[32]	No. 1	61	M	Yes	Yes	No	971 U/L	40.76 mg/L	NA	Bilateral multiple GGOs with diffuse interlobular septal thickening
[33]	No. 2	69	F	Yes	Yes	Yes	NA	NA	NA	Bilateral diffuse GGO
[34]	No. 1	70	F	Subfever	NA	Yes	947 U/L	3.28 mg/dL	5280 U/mL	A diffuse, minimal, nonspecific ground‐glass appearance in both lungs
[35]	No. 1	73	M	Intermittent fever	Yes	Yes	480 IU/L	NA	NA	Diffuse ground‐glass opacities, bilateral hilar calcified lymph nodes
[36]	No. 1	73	M	NA	NA	Yes	NA	NA	2098 U/mL	Multiple patchy GGO in the bilateral lungs and a solid lesion in the right lower lobe of the lung (approximately 2 cm)
[37]	No. 1	66	M	Yes	Yes	Yes	2726.3 U/L	56.6 mg/L	NA	GGOs in bilateral lung fields and mild pericardial effusion
[38]	No. 1	65	F	Intermittent fever		NA	281.25 U/L	44.93 mg/L	236 U/mL	Scattered subpleural GGO in both lungs
[39]	No. 1	68	M	Intermittent high fevers	Yes	Yes	2340 IU/L	13.4 mg/L	NA	Thickened interlobular septa, multiple micronodules and ground‐glass opacity patched in bilateral lungs
	No. 2	60	F	Intermittent high fevers	NA	Yes	1434 IU/L	79.9 mg/L	NA	Diffuse centrilobular nodules and ground‐glass opacity in the bilateral lungs, with local thinckened interlobular septa
	No. 3	69	F	Yes	Yes	Yes	1095 IU/L	88.5 mg/L	NA	Multiple patches, and GGO in bilateral lungs, with local interlobular septal thickening

Abbreviations: CRP, C‐reactive protein; F, female; GGN, ground‐glass nodule; GGO, ground‐glass opacity; LDH, lactate dehydrogenase; M, male; sIL2‐R, soluble interleukin‐2 receptor.

**TABLE 3:** Summary of reported IVLBCL with pulmonary involvement and without abnormal chest CT findings.

References	Case	Age	Sex	Clinical feature			Laboratory findings			FDG PET/CT findings
				Fever	Cough	Dyspnea	LDH	CRP	sIL2‐R	
[40]	No. 1	58	Female	40°C	Yes	Yes	570 IU/L	10.3 mg/dL	3699 U/mL	NA
[41]	No. 1	43	Male	Yes	Yes	Yes	Increased	NA	NA	Diffuse and dense FDG uptake of the lungs (SUVmax: 5.44)
[42]	No. 1	60	Male	39.8°C	NA	Yes	Increased	Increased	NA	Diffuse hypermetabolic bilateral pulmonary FDG uptake, greater than physiologic hepatic uptake
[43]	No. 1	41	Male	Yes	Yes	NA	Increased	NA	NA	Diffuse FDG uptake in and the bilateral lungs before treatment; PET showed disappearance of FDG uptake in the bilateral lungs after treatment
[44]	No. 1	58	Female	Yes	NA	NA	NA	NA	6774 pg/mL	A subsequent examination using FDG PET/CT showed a high uptake in the bilateral lung
[22]	No. 3	65	Male	No	Yes	Yes	886 IU/L	27 mg/L	NA	NA
[45]	No. 1	84	Male	NA	NA	NA	641 IU/L	NA	2238 U/mL	There was no abnormal accumulation of FDG in bilateral lung fields
[46]	No. 1	63	Male	Yes	NA	NA	520 U/L	NA	NA	Mild FDG activity in both lungs (SUVmax = 2.2)

Abbreviations: CRP, C‐reactive protein; FDG PET/CT, fluorodeoxyglucose positron emission tomography/computer tomography; LDH, lactate dehydrogenase; sIL2‐R, soluble interleukin‐2 receptor.

### 3.1 Clinical Features and Laboratory Findings

IVLBCL can manifest with fever, fatigue, weight loss, and other symptoms. Lung involvement may cause cough, dyspnea, and hypoxemia. However, clinical manifestations may be absent in some cases, such as in Case 2 and a previous case [45]. Laboratory findings frequently revealed elevated LDH and soluble interleukin‐2 receptor (sIL‐2R) levels, thrombocytopenia, and other abnormalities (Table 2). Moreover, a previous study found that sIL2R levels were significantly higher in patients with Asian‐variant IVLBCL than in those with non‐Asian‐variant IVLBCL [47].

### 3.2 Diagnostic Clues in Medical Imaging

GGO is typically defined as an area exhibiting hazy obscuration or increased attenuation in chest imaging. It results from the replacement of air by various factors, including fluid, collapsed airways, fibrotic tissue, or neoplastic growth. According to previous case reports and our case series of IVLBCL with pulmonary involvement, bilateral GGOs were the most common chest CT manifestations in these patients [29, 48]. A total of 35 patients (20 males and 15 females, aged 35–84 years) with pulmonary IVLBCL were reviewed based on previous case reports and three cases from our hospital. Among them, 21 cases (including Case 3) showed multiple or diffuse GGO infiltrations in both lungs (60.0%), 6 cases (including Cases 1 and 2) showed multiple GGOs with clear borders (17.1%), and no obvious abnormal chest CT findings were observed in eight cases (22.9%). This finding may be related to the pathological presentation of IVLBCL in the lungs, which typically involves widening of the interstitial space between the alveoli, expansion of capillaries and lymphoid tissue, and filling of the lumen of the bronchioles with lymphoid cells.

Although bilateral GGOs are frequently observed in IVLBCL patients, these findings lack specificity. FDG PET/CT scans can reveal high diffuse uptake of FDG in lesions of IVLBCL patients earlier, providing crucial diagnostic information such as lesion location and extent of IVLBCL involvement [49, 50]. It is important to note that other conditions, including acute respiratory distress syndrome, pulmonary contusion, and inflammatory or infectious pneumonitis, may also manifest as GGOs on CT scans, particularly in areas with increased FDG uptake. These conditions can be differentiated based on the patient's medical history and disease course [42]. Therefore, FDG PET/CT plays an important role in the diagnosis of IVLBCL.

In addition, some case reports demonstrated that some pulmonary IVLBCL patients could have no abnormality on chest CT, although some of them had fever and dyspnea (Table 3). However, diffuse FDG uptake is usually observed in the lungs. Diffuse FDG uptake without evident chest CT findings is called *hot lung*, which is quasi‐pathognomonic for pulmonary IVLBCL [41]. When pulmonary IVLBCL is highly suspected clinically but chest CT is normal, FDG PET/CT can provide valuable information. Additionally, *hot lung* may also indicate chemotherapy‐induced pneumonia or acute respiratory distress syndrome, which requires differentiation based on the patient's history [41, 42].

As a noninvasive imaging technique, FDG PET/CT has significant value in IVLBCL patients [47, 51]. FDG PET/CT also plays an important role in guiding biopsy sites and evaluating the extent of systemic involvement or response to chemotherapy after relapse [51]. Therefore, even if there are no obvious abnormalities in chest CT scans, FDG PET/CT can be considered to obtain more comprehensive diagnostic information non‐invasively for patients with fever of unknown origin, fatigue, elevated LDH levels, ineffective conventional anti‐inflammatory treatment, or those who are highly suspected of pulmonary IVLBCL.

In addition, it is crucial to recognize that diagnostic tests like FDG PET/CT and TBLB cannot detect all lesions with 100% accuracy. Therefore, the doctor's assessment and judgment play a vital role in the diagnostic process. One patient was diagnosed with pulmonary IVLBCL by TBLB without obvious abnormal chest CT and FDG PET/CT findings in a previous literature report [45]; there was focal FDG high uptake and no abnormal TBLB findings in Case 2.

## 4 Conclusions

IVLBCL should be considered in patients with symptoms such as fever of unknown origin, fatigue, elevated LDH levels, or hemophagocytic syndrome. In the diagnosis and monitoring of pulmonary IVLBCL, chest CT scans are indispensable. Bilateral GGOs are frequently observed and may show improvement following therapeutic intervention. Furthermore, GGOs may sometimes manifest as multiple, distinct GGNs with clear boundaries. Although chest CT scans may occasionally appear unremarkable, elevated FDG uptake in PET/CT imaging can suggest lung involvement. When FDG PET/CT yields positive results, prompt biopsy should be considered. Accurate diagnosis depends on the combination of H&E staining for morphological examination and IHC to verify B‐cell lineage and distinguish IVLBCL from other lymphoma types, thereby informing appropriate treatment approaches.

## Author Contributions

Conceptualization: Xinyi Gou, Libao Hu, and Jin Cheng. Methodology: Yuan Li and Jin Cheng. Software: Xinyi Gou. Data curation: Yinli Zhang and Yuan Li. Investigation: Xinyi Gou, Yinli Zhang, and Yuan Li. Validation: Xinyi Gou and Yinli Zhang. Formal analysis: Xinyi Gou. Supervision: Libao Hu and Jin Cheng. Funding acquisition: Libao Hu. Visualization: Xinyi Gou. Project administration: Jin Cheng and Nan Hong. Resources: Jin Cheng and Nan Hong. Writing – original draft: Xinyi Gou. Writing – review and editing: Xinyi Gou, Yinli Zhang, Libao Hu, Jin Cheng, and Nan Hong.

## Ethics Statement

This retrospective study was approved by the Ethics Review Committee of the Peking University People's Hospital (no. 2022PHB276‐001). The authors are accountable for all aspects of the work, ensuring that questions related to the accuracy or integrity of any part of the work are appropriately investigated and resolved. All procedures performed in studies involving human participants were in accordance with the ethical standards of the institutional and/or national research committee(s) and with the Helsinki Declaration (as revised in 2013). Written informed consent was obtained from all participants, or if participants were under 16 years of age, from a parent and/or legal guardian.

## Consent

All procedures were discussed with the patients, and they gave their full consent in writing to the present publication and accompanying images.

## Conflicts of Interest

The authors declare no conflicts of interest.

## Data Availability

The data that support the findings of this study are available on request from the corresponding author. The data are not publicly available due to privacy or ethical restrictions.
